# Death Receptors in the Selective Degeneration of Motoneurons in Amyotrophic Lateral Sclerosis

**DOI:** 10.1155/2013/746845

**Published:** 2013-07-16

**Authors:** Julianne Aebischer, Nathalie Bernard-Marissal, Brigitte Pettmann, Cédric Raoul

**Affiliations:** ^1^Inserm-Avenir team, The Mediterranean Institute of Neurobiology (INMED), 13288 Marseille, France; ^2^Neurodegenerative Studies Laboratory, Brain Mind Institute, The Swiss Federal Institute of Technology Lausanne (EPFL), CH-1015 Lausanne, Switzerland; ^3^Department of Medical Genetics, University of Lausanne, CH-1005 Lausanne, Switzerland; ^4^The Mediterranean Institute of Neurobiology (INMED), Inserm UMR901, 13288 Marseille, France; ^5^The Neuroscience Institute Montpellier (INM), INSERM UMR1051, Saint Eloi Hospital, 34091 Montpellier, France

## Abstract

While studies on death receptors have long been restricted to immune cells, the last decade has provided a strong body of evidence for their implication in neuronal death and hence neurodegenerative disorders such as amyotrophic lateral sclerosis (ALS). ALS is a fatal paralytic disorder that primarily affects motoneurons in the brain and spinal cord. A neuroinflammatory process, associated with astrocyte and microglial activation as well as infiltration of immune cells, accompanies motoneuron degeneration and supports the contribution of non-cell-autonomous mechanisms in the disease. Hallmarks of Fas, TNFR, LT-*β*R, and p75^NTR^ signaling have been observed in both animal models and ALS patients. This review summarizes to date knowledge of the role of death receptors in ALS and the link existing between the selective loss of motoneurons and neuroinflammation. It further suggests how this recent evidence could be included in an ultimate multiapproach to treat patients.

## 1. Introduction

The name of “death receptors” is associated with the tumor necrosis factor receptor superfamily (TNFRSF) of transmembrane proteins whose first shown function was to induce apoptosis in subtypes of immune cells (for review see [1]). For example, Fas (CD95, Apo1) was identified in 1989 as a receptor being activated during the negative and positive selection of T cells [2, 3]. Since this pioneer work, death receptor activation has been evidenced in a variety of nonimmune cells and shown to induce, besides apoptosis, a number of nonapoptotic events including regulation of cell proliferation and differentiation, chemokine production, inflammatory responses, and tumor-promoting activities [4]. 

In the nervous system, most death receptors are expressed by neurons as well as by glial cells during development. Nevertheless, while activation of Fas or TNFR induces death of embryonic nervous cells *in vitro* [5–7], there is still little evidence about their implication in developmental cell death. On the contrary, a growing body of data has demonstrated the involvement of death receptors in neurodegenerative diseases. Depending on models and experimental conditions, studies have confirmed the proapoptotic effects resulting from the activation of death receptors in Alzheimer's disease [8, 9] or in Parkinson's disease [10] but have also pointed out their nonapoptotic functions in cell protection in Parkinson's disease [11, 12]. Probably the strongest evidence for the involvement of death receptors in neurodegenerative diseases comes from studies on amyotrophic lateral sclerosis (ALS). ALS is a fatal and incurable neurodegenerative disorder characterized by the progressive loss of both upper and lower motoneurons. ALS has a complex multifactorial aetiology as reflected by the large predominance of sporadic forms of the disease. Among the 10% of familial cases, about 20% are caused by dominant mutations in the *superoxide dismutase-1* (*SOD1*) gene [13]. Mice expressing human SOD1 mutations develop a motor syndrome with features of the human disease and have greatly contributed to our comprehension of the pathogenic processes [14]. Accumulating evidence has emerged for both cell- and non-cell-autonomous effects of mutated SOD1 mutant in the pathogenic process. Notably, the depletion of mutated SOD1 in motoneurons significantly delays the onset and early phase of the disease [15], whereas a non-cell-autonomous toxic action of mutated SOD1 in astrocytes and microglia is determinant for disease onset and/or progression [16, 17]. Finally, blood-derived immune cells, which infiltrate the central nervous system (CNS) early in the disease, have been proposed to contribute to the neuroinflammatory process, which typifies ALS. Thus, the identification of the detrimental non-cell-autonomous effectors is a crucial step to eventually design pertinent therapeutic approaches. This review focuses on the last decade evidence for a role of four members of the TNFRSF, Fas, TNFR1, LT-*β*R, and p75^NTR^ in the death of motoneurons in ALS and how this new knowledge could be the basis of new therapeutic approaches in ALS.

## 2. The Fas Death Pathway 

Fas/CD95 belongs to the TNFRSF and is known for its capacity to induce apoptosis in various sensitive cells. Binding of Fas ligand (FasL/CD95L) causes a conformational change in the preassembled tetrameric Fas receptor, which leads to the recruitment of the Fas-associated protein with death domain (FADD), to form the death-inducing signaling complex [18]. The formation of this complex leads to the recruitment and activation of caspase-8 or -10, which depending on the cell type, can either promote the cleavage of the effector caspase-3 directly and/or indirectly by cleaving the BH3-only family member Bid [18]. This will eventually induce the release of cytochrome c from the mitochondria and the subsequent cleavage of caspase-9, an initiator caspase that activates the executioner caspase-3 [19]. Another death signaling mechanism triggered by Fas/FasL interaction includes the recruitment of the adaptor protein, death associated protein 6 (Daxx), and the subsequent activation of various MAP kinases, such as apoptosis signal-regulating kinase 1 (ASK-1), p38 kinase, or the Jun N-Terminal kinase [20–22].

Both Fas and FasL are broadly expressed proteins, and within the CNS, they were shown to be expressed by neurons and glial cells both during development and all the way through adult life [23–28]. During development, motoneurons purified at the embryonic stage at which they enter the naturally occurring cell death period strongly express Fas and FasL. *In vitro* studies have further shown that the deprivation of neurotrophic factors triggers death of embryonic motoneurons in a Fas-dependent manner [6, 7]. There is however still little evidence for a contribution of Fas in the elimination of motoneurons during development, since the incomplete loss-of-function mutants for Fas and FasL (*lymphoproliferative, lpr *and *generalized lymphoproliferative* disease, and *gld *mice, resp.) have normal numbers of motoneurons at birth [7, 28]. However, the number of surviving motoneurons in neonatal transgenic mice overexpressing a dominant negative form of FADD, which abrogates the Fas-FADD signaling branch, was shown to be twofold higher after transection of the facial nerve. These results reveal that besides its potential role in development, Fas may also be implicated in pathological degeneration of motoneurons [7].

**Figure 1:**
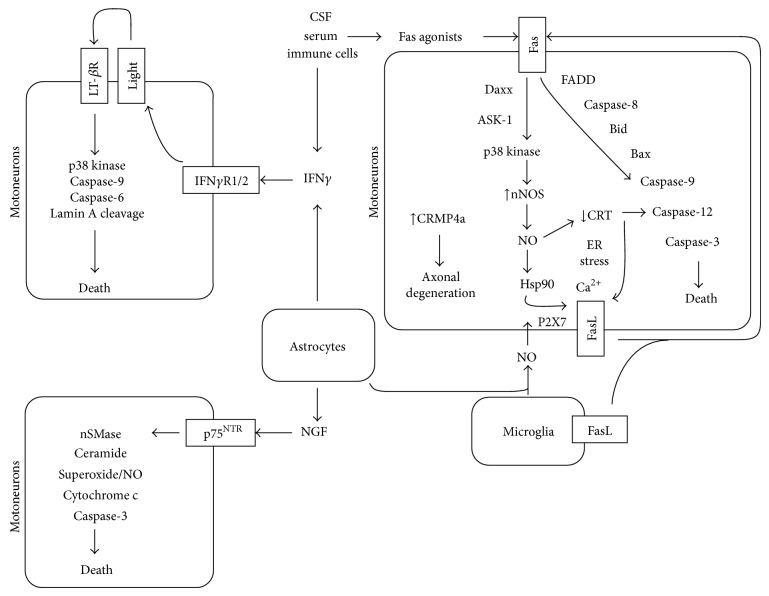
Active killing of motoneurons through death receptors under pathological context. Fas activation induces a motoneuron-restricted signaling pathway, in which both Fas-Daxx and Fas-FADD branches act synergistically to execute the death program. The production of NO is an obligatory step of the Fas pathway that leads to the downregulation of CRT, which promotes ER stress and upregulation of FasL that in turn activates Fas. Mobilization of FasL to the plasma membrane was also shown to occur following the stimulation of P2X7 receptor by nitrated Hsp90. CRMP4a ALS-linked SOD1 sensitizes motoneurons to this Fas/NO feedback loop. The entry in the amplification loop can be achieved by exogenous NO or Fas agonists (i.e., FasL or circulating agonistic anti-Fas antibodies) that can originate from motoneuron environment. IFN*γ* produced by mutant astrocytes promotes the engagement of LT-*β*R pathway by increasing levels of LIGHT in motoneurons. LT-*β*R-mediated death of motoneurons implicates p38 kinase, caspase-9 and -6, and lamin A cleavage but occurs independently of caspase-8, cytochrome c release, and the caspase-3/-7 pathway. Immune cells, serum, and cerebrospinal fluid may represent another source for IFN*γ*. NGF produced by activated astrocytes triggers death of motoneurons following engagement of p75^NTR^, which become reexpressed in pathological condition. p75^NTR^-induced death of motoneurons involves activation of nSMase, production of ceramide, formation of reactive oxygen and nitrogen species, the release of cytochrome c from the mitochondria, and activation of caspase-3. Regarding their own proper characteristics, vulnerable motoneurons would be more susceptible to this non-cell-autonomous effect.

Primary culture of embryonic motoneurons has greatly contributed to the understanding of Fas death signaling. Indeed, the exogenous activation of Fas by either soluble FasL or an agonistic anti-Fas antibody leads to the death of up to 50% of cultured motoneurons, offering a means to study events downstream of Fas [6, 29]. In addition to the classical Fas-signaling pathway, involving FADD recruitment, a parallel signaling pathway implicating the recruitment of the proteins Daxx, ASK-1, and p38 kinase and the atypical production of nitric oxide (NO) is necessary to induce motoneuron death, following Fas activation (Figure 1) [29]. Interestingly, motoneurons expressing ALS-linked mutated SOD1 present an exacerbated sensitivity to the Fas death pathway. However, mutant SOD1 does not increase the killing effect of Fas on other Fas-sensitive cell types such as cortical neurons or thymocytes. Furthermore, the Daxx/ASK/p38 kinase/NO pathway may act as an amplification loop, where the production of NO leads to the expression of sFasL, which in turn activates Fas [27]. An interesting mechanism linking the production of NO and FasL expression has been recently proposed. The nitration of the heat-shock protein 90 (Hsp90), following the formation of peroxynitrite by the reaction of NO and superoxide, was shown to mobilize FasL at the plasma membrane following stimulation of the Ca^2+^-permeable cationic channel P2X7 [30]. *In vivo*, FasL has been shown to be upregulated in motoneurons at a presymptomatic stage of the disease in *SOD*1^*G*93*A*^ mice, and this upregulation is reduced when the SOD1 mutant mice are expressing a dominant negative form of Daxx. All together, this evidence suggests that a motoneuron-restricted exacerbation to Fas activation, conferred by ALS-linked mutated SOD1, might contribute to the selective degeneration of motoneurons in ALS.

Recent studies on the Fas/NO pathway have shed light on two other proteins, calreticulin (CRT) and the collapsing response protein 4 (CRMP4a), that are implicated in the death of mutant SOD1 motoneurons specifically (Figure 1) [31, 32]. Authors observed that Fas or NO treatment of cultured embryonic motoneurons leads to a decrease in CRT expression and to an increase in CRMP4a levels. A change in the expression of these two proteins is sufficient to induce death of motoneurons. On one hand, the decrease in CRT expression leads to an upregulation of ER stress sensors combined with disturbances in calcium homeostasis; two events that eventually trigger death of motoneurons expressing mutated SOD1. Conversely, CRT overexpression was able to rescue mutant motoneurons from Fas/NO-induced death. *In vivo*, as it is observed for ER stress, CRT downregulation in motoneurons precedes muscle denervation and is restricted to vulnerable motoneuron populations [31]. On the other hand, the RNA interference-mediated silencing of CRMP4a is protective against NO-induced toxicity of cultured motoneurons. Furthermore, an increase in CRMP4a levels can be detected *in vivo* in a proportion of motoneurons in the spinal cord of *SOD*1^*G*93*A*^ mice at presymptomatic stage [32]. Consistently, the viral-induced overexpression of CRMP4a is sufficient to trigger the denervation of motor endplates and motoneuron degeneration. Further experiments are required to explore the contribution of both CRT and CRMP4a in the disease process, which in case would enlarge the number of potential therapeutic targets for therapies linked to Fas/NO-induced motoneuron death. Besides the disturbances observed in the expression of CRT and CRMP4a, other components of the Fas/NO pathway, such as p38 kinase, ASK1, or neuronal nitric oxide synthase, were shown to be activated or increased in the spinal cord of ALS mouse models starting at presymptomatic stages of the disease [27, 33–40].

One aspect, which remains unclear, is the origin of FasL or NO in the pathological context of ALS. In the spinal cord of patients and ALS models, reactive astrocytes show an upregulation of nitric oxide synthase and display markers of oxidative stress [41–43]. Interestingly, in cocultures of astrocytes with motoneurons, astrocytes expressing SOD1 mutations were shown to kill motoneurons by secreting neurotoxic factors [44, 45]. NO has been proposed to potentiate the neurotoxicity of astrocytes expressing ALS-linked mutated SOD1 toward motoneurons [46]. We can propose that reactive mutant astrocytes release NO and thereby contribute to the death of motoneurons by initiating the Fas/FasL death pathway. However, Nagai and colleagues have shown that the inhibition of FasL by a soluble Fas decoy fails to improve the survival of embryonic mouse stem cell-derived motoneurons co-cultured with *SOD*1^*G*93*A*^ astrocytes [45]. Survival assays have also been performed on microglia/motoneuron co-culture systems. When compared to astrocytes, microglia cells expressing mutated SOD1 have only a modest effect on motoneuron survival [45, 47]. Once activated *in vitro*, microglial cells may however start to secrete toxic factors such as NO and FasL and therefore remain a potential source of Fas/NO in ALS [47–49]. Additional experiments are however needed to determine if astrocytes in a concerted action with microglial cells are involved in triggering death of motoneuron through the activation of the Fas/NO death pathway.

As agonistic anti-Fas antibodies have been detected in the serum of ALS patients [50, 51], a last potential source of FasL/NO could be immune cells. Indeed, increased numbers of CD8^+^ and CD4^+^ T cells as well as natural killer (NK) cells are found in the spinal cord of mutant SOD1 mice beginning at presymptomatic stages of the disease and increasing with its progression [52]. In the CNS, CD8^+^ T cells can induce apoptosis of target cells through the Fas/FasL, perforin/granzyme system, and IFN*γ* [53]. IFN*γ* that can be released by CD4^+^ T helper 1 (Th1) has been recently proposed as a pertinent therapeutic candidate using a system mathematical model [54]. Although several studies have pointed out the implication of astrocytes and microglia cells in the process of neuroinflammation in ALS, future studies are needed to clarify the role of immune cells in triggering the Fas/NO or IFN*γ* death pathway implicated in motoneuron degeneration.

The functional implication of the Fas death pathway in ALS pathogenesis was further supported by studying ALS mice with a partial deletion of FasL. Indeed, *gld* mutant mice expressing ALS-linked *SOD*1^*G*93*A*^ mutation show improved motor behaviour and increased motoneuron survival when compared to *SOD*1^*G*93*A*^ littermates [55]. Moreover, the intrathecal delivery of small interfering RNA directed against Fas at disease onset delays the progression of the disease in ALS mice [56]. Additional evidence, though indirect, suggests that Fas contributes to ALS pathogenesis. Matrix metalloproteinases (MMPs) have been shown to cleave the membrane-bound FasL to release its soluble form. The genetic deletion of MMP-9 has controversial effects on mutant SOD1 mice: in one study, it led to the reduction of FasL and TNF*α* immunoreactivity in the spinal cord and extended survival of ALS mice [57], but in another, it exacerbated the motor symptoms [58]. Administration of a broad-spectrum inhibitor of MMPs had a positive impact on the lifespan of treated mutant SOD1 mice [59]. Further research is needed to complete our understanding of MMPs in ALS pathogenesis and evaluate their potential as therapeutic candidates.

## 3. The IFN**γ**-LIGHT/LT-**β**R Death Pathway

LIGHT (TNFSF14, CD258), which stands for “lymphotoxin-related inducible ligand that competes for glycoprotein D binding to herpes virus entry mediator on T cells,” is a member of the TNFSF, which is mainly expressed on the surface of immature dendritic cells, NK cells, and activated lymphocytes and monocytes, and which participates in innate and adaptive immune responses [60–62]. Produced as a type II transmembrane protein, LIGHT can be cleaved by proteolysis into a soluble form and acts as a homotrimer to engage two distinct membrane-bound receptors, the lymphotoxin-*β* receptor (LT-*β*R, TNFRSF3, and CD18) and the receptor known as Herpes virus entry mediator (HVEM, TNFRSF14, and CD270) [63, 64]. Even though LT-*β*R and HVEM lack an actual death domain, LIGHT was shown to induce moderate apoptosis of numerous tumor cell lines with a characteristically slow kinetic upon activation of LT-*β*R. Interestingly, this death-inducing property of LIGHT/LT-*β*R interaction depends on the presence of IFN*γ* [65–68]. The expression of LIGHT was also observed in the CNS, where both of its receptors, LT-*β*R and HVEM, are also present [69, 70]. In the CNS, LIGHT was shown to negatively regulate axon growth of nodose sensory neurons by activating the HVEM receptor [69].

Recently, LIGHT has been shown to contribute to ALS pathogenesis. LIGHT has a deleterious effect on motoneuron survival, whereas at first sight this ligand does not affect the survival of other neuronal types. Indeed, cultured embryonic motoneurons are dose-dependently sensitive to soluble LIGHT, with a plateau being reached 48 hours after treatment at 50% of motoneuron loss. Even though cultured motoneurons express both of LIGHT's receptors, it is the engagement of the LT-*β*R that is necessary and sufficient to induce their apoptosis. The intracellular signaling mechanism triggered by LIGHT greatly differs from that observed for FasL. In contrary to FasL, LIGHT-induced death of motoneurons does not depend on the activation of caspase-8, nor does it induce the phosphorylation of p38 kinase or lead to the release of cytochrome c from the mitochondria. And even though the cleavage of caspase-9 plays a central role in both pathways, apoptotic degeneration of motoneurons following LIGHT treatment is mediated by the effector caspase-6, without implication of caspase-3 (Figure 1). Interestingly, a combination of both FasL and LIGHT triggers up to 70% of motoneuron loss [71]. One possible explanation for the additive killing effect of LIGHT and FasL is that they target different populations of motoneurons, a hypothesis that still needs to be investigated. This might be of particular interest when considering therapeutic approaches for ALS, since it might indicate that targeting both death pathways at the same time could have a greater impact on motoneuron survival.

As it has been observed for tumor cell lines, LIGHT-induced death of motoneurons is also tightly regulated by the proinflammatory cytokine IFN*γ* [71]. In fact, a close interplay exists between LIGHT and IFN*γ*. Whilst the presence of the cytokine specifically potentiates the effect of LIGHT, IFN*γ* by itself may also trigger a dose-dependent death of motoneurons. Indeed, binding of IFN*γ* to its receptor complex IFN*γ*RI/IFN*γ*RII on motoneuron cell surface leads to an upregulation of LIGHT expression, followed by death of motoneurons through activation of the LT-*β*R. These results outline the important regulatory function of IFN*γ* in the death pathway triggered by LIGHT/LT-*β*R interaction.

Interestingly, several studies have now shown that IFN*γ* levels increase during the course of the disease in the spinal cord tissue, cerebrospinal fluid, and serum of ALS mouse models and patients [72–75]. Whilst Th1, cytotoxic T, and NK cells are all potential well-known sources of IFN*γ*, Aebischer et al. have recently shown that astrocytes also contribute to IFN*γ* upregulation in ALS spinal cords [71, 76]. Indeed, primary astrocyte cultures from *SOD*1^*G*93*A*^ mice or rats show elevated levels of IFN*γ* production and secretion when compared to wild-type cells. Furthermore, IFN*γ* has recently been identified as a contributor to the astrocyte-derived neurotoxicity in the astrocyte/motoneuron cocultures, with motoneurons being rescued from *SOD*1^*G*93*A*^ astrocyte toxicity when treated with molecules that interfere with either IFN*γ* or the LIGHT/LT-*β*R pathway.

In contrary to what is observed for FasL, ALS mutations do not lead to an increased sensitivity of motoneurons to LIGHT and/or IFN*γ*, but it is instead the aberrant secretion of IFN*γ* by mutant astrocytes and possibly other cell types that links the LIGHT/LT-*β*R pathway to motoneuron degeneration in this disease. In fact, murine motoneurons *in vivo* constitutively express both LIGHT and LT-*β*R with no difference being observed between wild-type and *SOD*1^*G*93*A*^ animals during the course of the disease. An increased expression of IFN*γ* is however observed in *SOD*1^*G*93*A*^ mice beginning at disease onset, and IFN*γ* levels continue to be elevated during the progression phase of the disease, with both activated astrocytes and motoneurons being immunoreactive for IFN*γ*.

Evidence for the functional implication of this IFN*γ*-induced and LIGHT/LT-*β*R-mediated death pathway in ALS pathology comes from the study of double transgenic mice. Whereas the genetic deletion of *Light* in *SOD*1^*G*93*A*^ mice does not affect disease onset, it significantly retards progression and extends lifespan of ALS mice. These *in vivo* data point more precisely towards a contribution of LIGHT/LT-*β*R interaction to the progression of the disease, where activated astrocytes seem to play a pivotal role by expressing IFN*γ*, the key trigger factor of this death pathway.

Evidence for an implication of this LIGHT/LT-*β*R death pathway in ALS pathology has further been provided by the analysis of tissues obtained from human patients. LIGHT and LT-*β*R are constitutively expressed in large ventral horn neurons in both postmortem sporadic ALS patients and non-ALS controls. However, a significant increase in the levels of LIGHT, but not LT-*β*R, was observed in the spinal cord of ALS patients when compared to controls. In accordance with other studies, Aebischer et al. also documented an increase in IFN*γ* levels in spinal cords of ALS patients. As observed in the *SOD*1^*G*93*A*^ mouse model, IFN*γ* signals were detected in large ventral horn neurons as well as nonneuronal cells that resemble astrocytes and immune cells [77]. This study not only confirms the observation made in the *SOD*1^*G*93*A*^ mouse model on the implication of the IFN*γ*/LIGHT/LT-*β*R death pathway in ALS, but also it further extrapolates these findings to a larger number of clinical cases, with sporadic ALS patients showing signs of activation of this death pathway. Interestingly, a positive correlation has recently been observed between rapid progression rates of the disease and postmortem spinal IFN*γ* levels in ALS patients [73].

Besides its direct contribution to the LIGHT/LT-*β*R-mediated toxicity of astrocytes towards motoneurons, IFN*γ* may also promote the neuroinflammatory status observed during ALS. Studies performed on primary cultures of wild-type astrocytes have, for example, shown that this cytokine may induce the expression of NO, which may in turn lead to the activation of astrocytes, therewith reinforcing their toxic properties [78]. It is also possible that this IFN*γ*-triggered NO production may directly affect motoneuron survival by activation of the Fas death pathway. Alternatively, IFN*γ* may also modify the reactive status of microglial cells and therewith potentially participate in other pathogenic processes implicated in ALS [79]. IFN*γ* may, for example, directly promote the secretion of FasL by activated microglial cells, pointing out the possible link that exists between IFN*γ* and various death pathways [80]. 

## 4. The TNF***α***/TNFR1 Pathway

TNF*α* is a homotrimeric type II protein that can be released in the CNS by astrocytes, microglial cells, or some neuronal cells. This proinflammatory cytokine is active either as a membrane-bound or soluble form to signal via two distinct receptors of the TNFRSF, both constitutively expressed by neurons and glial cells: TNF receptor 1 (TNFR1) and TNFR2, with only the former harbouring a cytoplasmic death domain [81]. Similarly to the classical Fas death pathway, TNF*α* binding to TNFR1 leads to the recruitment of the adaptor proteins TRADD and FADD and the subsequent activation of a caspase cascade that depends on the release of cytochrome c from the mitochondria. By the activation of this death pathway, TNF*α* triggers death of primary embryonic motoneurons, with a plateau being reached upon 50% of motoneuron loss, as observed for FasL and LIGHT [7, 71]. A simultaneous activation of Fas and TNFR1 does not however increase the percentage of motoneuron loss, supporting the hypothesis that a population of motoneurons is intrinsically resistant to the activation of this intracellular death pathway [7].

An increase in the expression of TNF*α* as well as both of its receptors has been documented in mutant SOD1 mice [38, 74, 82–84]. The increase in TNF*α* is observed before the appearance of obvious behavioural deficits in these mice and is closely associated with disease progression. Elevated TNF*α* levels have also been detected in the plasma of ALS patients [85, 86]. So far however, no experimental evidence allows establishing a conclusive link between the aberrant expression of TNF*α* and the degeneration of motoneurons in the pathological context of ALS. Indeed, the genetic ablation of TNF*α* in mice expressing mutated SOD1 does not alter the course of the disease, nor does it improve the lifespan or these animals [87]. It is however not excluded that TNF*α* plays a role in the inflammatory component of the disease. Indeed, primary astrocyte cultures from *SOD*1^*G*93*A*^ neonates express more TNF*α* in basal state when compared to nontransgenic astrocytes, and they start to overexpress TNF*α* when challenged with IFN*γ* or TNF*α* itself [74]. Similarly, in response to stimulation by lipopolysaccharide, *SOD*1^*G*93*A*^ microglial cell *in vitro* also produces TNF*α*. This cytokine may furthermore participate in maintaining the reactive status of glial cells, therewith promoting their release of proinflammatory mediators, which contribute to the microglia-derived toxicity towards motoneurons [47, 88, 89]. 

## 5. The NGF/p75^**NTR**^ Pathway

Nerve growth factor (NGF) is a neurotrophin that has a high binding affinity to the tropomyosin kinase-related tyrosine receptor kinase A (TrkA) and a lower affinity for the p75^NTR^ death receptor of the TNFRSF. Interestingly, the interaction of NGF with p75^NTR^ in the presence of TrkA promotes survival and neurite outgrowth, whereas in the absence of TrkA activation of p75^NTR^ may initiate a death signal [90]. 

Under normal conditions, motoneurons express neither of these two receptors and are therefore not sensitive to NGF. During the developmental period of programmed cell death, motoneurons however express p75^NTR^, and even though expression of this receptor is lost afterwards, it may be reinitiated in axotomized motoneurons [91, 92]. It has further been documented that activation of the p75^NTR^ receptor is actually implicated in the loss of motoneurons observed after facial nerve lesion in mice [93].

Reexpression of p75^NTR^ has also been detected in motoneurons of both ALS mice and patients [94, 95]. NGF-induced death has been shown to rely on the activation of neutral sphingomyelinase (nSMase), production of ceramide, and the generation of NO and mitochondrial superoxide, which is accompanied by the formation of nitrotyrosine protein adducts, as well as cytochrome c release and caspase-3 activation [96, 97] (Figure 1). Interestingly, *in vitro* studies have also shown that activation of astrocytes by the fibroblast growth factor-1 (FGF-1) or by reactive nitrogen species including NO or peroxynitrite leads to the production of NGF that induces death of motoneurons in a p75^NTR^-dependent manner [96, 98]. Furthermore, in the absence of NO, NGF is selectively toxic for motoneurons expressing mutated SOD1 [97]. Evidence for the functional implication of the p75^NTR^ death receptor in ALS comes from two independent studies, where a partial or complete deletion of p75^NTR^ expression was shown to positively affect the survival of mutant SOD1 mice [99, 100]. However, no significant protection of motoneurons was observed, which suggested an indirect role of p75^NTR^ on motoneuron degeneration.

## 6. Therapeutic Implication

The ultimate aim of a therapeutic approach for ALS is to interfere with the progressive degeneration of motoneurons and to halt the loss of motor units and therewith the installation of fatal paralysis. Removal of the ALS-causative gene might be the most straightforward approach [101, 102], but it is however still technically very challenging and applicable to only a small subset of clinical cases, namely, familial ALS patients. Since ALS is a multifactorial disease, targeting single common downstream motoneuron-intrinsic contributors to disease might not be sufficient to prevent their degeneration. This could explain why clinical trials have so far given disappointing results. This especially holds true when considering that ALS is a non-cell-autonomous disease where surrounding nonneuronal cells actively participate in motoneuron damage. Indeed, as already discussed, activated astrocytes and microglial cells as well as infiltrating immune cells release cytokines and other toxic compounds that can either directly trigger motoneuron degeneration or consolidate the deleterious inflammatory status of their neighboring cells. Targeting neuroinflammation might therefore be an interesting therapeutic approach for this disease, especially since it is observed in both familial and sporadic ALS patients, and it is closely associated with progression of the disease [103]. To date, several studies have aimed at targeting neuroinflammation by suppressing activation of glial cells but have so far shown disappointing clinical results. The thalidomide derivative, lenalidomide, which shows anti-inflammatory properties by inhibiting the expression of TNF*α* and other cytokines, improves motor performance of *SOD*1^*G*93*A*^ mice and extends their survival, even when treatment begins with symptom onset [104, 105]. When administered to ALS patients, tolerable doses of thalidomide did not however show beneficial therapeutic effects, nor did they improve the cytokine profile of these patients, but on contrary, treatment was associated with adverse side effects [106, 107]. Similarly, minocycline, which is a second-generation tetracycline known for its anti-inflammatory properties to suppress macrophage and microglial activations also delayed disease onset and extended the survival of ALS mice but actually had a harmful effect on ALS patients and even accelerated their rate of deterioration [108–110]. The same type of observation was made when using celecoxib to inhibit cyclooxygenase 2, an enzyme that catalyzes the synthesis of prostaglandins and also triggers the production of various inflammatory cytokines. Whereas celecoxib protected motoneurons and prolonged survival of ALS mice, it did not show any therapeutic effects in ALS patients [111, 112]. Even though all these clinical trials have given disappointing results, it remains unclear whether it is a matter of the compound used (thalidomide in ALS patients versus lenalidomide in the mouse model), a wrong dosage of the drug administrated, or a result of the discrepancy that exists between the animal model and ALS patients.

One aspect that has to be taken in consideration is that inflammation is likely to play a dual role in the pathogenic process of ALS, since activated astrocytes and microglial cells may on one hand promote neuronal death, but on the other hand they can also preserve motoneuron survival by releasing anti-inflammatory cytokines, neurotrophins, or growth factors [113–115]. So even though neuroinflammation seems to be a promising site of intervention when considering therapies for ALS, it is important to design novel strategies to specifically target its deleterious branch. Interfering with death pathways triggered by the activation of death receptors might be one way to combat the downstream deleterious effect of neuroinflammation. Indeed, ALS mice that are deficient for *FasL* or *LIGHT* show improved survival of motoneurons and increased lifespan compared to their *SOD*1^*G*93*A*^ littermates [55, 71]. A therapeutic approach targeting both of these pathways at the same time might even show improved beneficial outcome, since the two pathways have an additive effect on motoneuron death *in vitro* and seem to target different populations of motoneurons [71]. Another approach could be to promote the secretion of anti-inflammatory cytokines while inhibiting the release of their proinflammatory counterparts. Recently, a study has analyzed the effect of various cytokines on disease progression using a mathematical model of a cell-cell communication network in ALS. They concluded that increasing the level of the anti-inflammatory cytokine interleukin (IL)-6 could have a beneficial impact on life expectancy by elevating the levels of IL-4 and that decreasing the expression of IFN*γ* could be an alternative therapeutic approach [54]. Indeed, IFN*γ* seems to play various central roles in the disease, and blocking the expression of this cytokine may therefore not only prevent the activation of the harmful arm of inflammation but also inhibit the motoneuron death pathway that depends on LIGHT and LT-*β*R. On top of that, this approach may also indirectly limit the activation of the FasL-induced apoptotic pathway, which contributes as well to motoneuron degeneration. Supporting this idea is the fact that a positive correlation has recently been observed between rapid progression rates of the disease and post-mortem spinal IFN*γ* levels in ALS patients [73]. The most interesting option for future therapeutic approaches may however still be to attack the disease from different angles.

## 7. Concluding Remarks

The presence of reactive astrocytes, activated microglia, and immune cells, as well as proinflammatory cytokines and reactive nitrogen or oxygen species at the damage site is common to most neurodegenerative diseases [116]. Therefore, such indiscriminating cellular and molecular mechanisms of neuroinflammation cannot likely determine the selectivity of motoneuron degeneration in ALS. One explanation might be that intrinsic features dictating the interpretation of these extrinsic signals are responsible for the selective vulnerability of motoneuron in this pathological context. The intrinsic feature of motoneurons to external death signals can be magnified by the presence of an ALS-associated gene product, as we showed with the exacerbated susceptibility of motoneurons expressing mutated SOD1 to FasL and NGF. This supports the idea that a minute and sustained accumulation of apoptotic signals leads to the progressive and selective degeneration of motoneurons. Another appealing hypothesis relies on a combinatory action of extrinsic signals. IFN*γ* and LIGHT, as well as NGF and NO, have been demonstrated to act synergistically to trigger motoneuron death. This suggests that a combinatorial diversity of inflammatory signals can also account for the specific elimination of motoneurons in ALS. We have stressed out that the selective neuronal vulnerability to extrinsic signals may therefore implicate several nonexclusive mechanisms acting through death receptors. Therefore, novel therapeutic strategies will have to answer the challenge of the functional diversity made by the combination of neuroinflammatory information.

## References

[1] Ashkenazi A. (2002). Targeting death and decoy receptors of the tumour-necrosis factor superfamily. *Nature Reviews Cancer*.

[2] Yonehara S., Ishii A., Yonehara M. (1989). A cell-killing monoclonal antibody (anti-Fas) to a cell surface antigen co-downregulated with the receptor of tumor necrosis factor. *Journal of Experimental Medicine*.

[3] Trauth B. C., Klas C., Peters A. M. J. (1989). Monoclonal antibody-mediated tumor regression by induction of apoptosis. *Science*.

[4] Peter M. E., Budd R. C., Desbarats J. (2007). The CD95 receptor: apoptosis revisited. *Cell*.

[5] Aebischer J., Sturny R., Andrieu D. (2011). Necdin protects embryonic motoneurons from programmed cell death. *PLoS ONE*.

[6] Raoul C., Henderson C. E., Pettmann B. (1999). Programmed cell death of embryonic motoneurons triggered through the Fas death receptor. *Journal of Cell Biology*.

[7] Ugolini G., Raoul C., Ferri A. (2003). Fas/tumor necrosis factor receptor death signaling is required for axotomy-induced death of motoneurons in vivo. *The Journal of Neuroscience*.

[8] He P., Zhong Z., Lindholm K. (2007). Deletion of tumor necrosis factor death receptor inhibits amyloid *β* generation and prevents learning and memory deficits in Alzheimer's mice. *Journal of Cell Biology*.

[9] Yamamoto M., Kiyota T., Horiba M. (2007). Interferon-*γ* and tumor necrosis factor-*α* regulate amyloid-*β* plaque deposition and *β*-secretase expression in Swedish mutant APP transgenic mice. *American Journal of Pathology*.

[10] Hayley S., Crocker S. J., Smith P. D. (2004). Regulation of dopaminergic loss by Fas in a 1-methyl-4-phenyl-1, 2, 3, 6-tetrahydropyridine model of Parkinson's disease. *The Journal of Neuroscience*.

[11] Landau A. M., Luk K. C., Jones M. (2005). Defective Fas expression exacerbates neurotoxicity in a model of Parkinson's disease. *Journal of Experimental Medicine*.

[12] Desbarats J., Birge R. B., Mimouni-Rongy M., Weinstein D. E., Palerme J., Newell M. K. (2003). Fas engagement induces neurite growth through ERK activation and p35 upregulation. *Nature Cell Biology*.

[13] Rosen D. R., Siddique T., Patterson D. (1993). Mutations in Cu/Zn superoxide dismutase gene are associated with familial amyotrophic lateral sclerosis. *Nature*.

[14] Kanning K. C., Kaplan A., Henderson C. E. (2010). Motor neuron diversity in development and disease. *Annual Review of Neuroscience*.

[15] Wang L., Sharma K., Deng H. (2008). Restricted expression of mutant SOD1 in spinal motor neurons and interneurons induces motor neuron pathology. *Neurobiology of Disease*.

[16] Ilieva H., Polymenidou M., Cleveland D. W. (2009). Non-cell autonomous toxicity in neurodegenerative disorders: ALS and beyond. *Journal of Cell Biology*.

[17] Wang L., Gutmann D. H., Roos R. P. (2011). Astrocyte loss of mutant SOD1 delays ALS disease onset and progression in G85R transgenic mice. *Human Molecular Genetics*.

[18] Lavrik I. N., Krammer P. H. (2012). Regulation of CD95/Fas signaling at the DISC. *Cell Death and Differentiation*.

[19] Scaffidi C., Fulda S., Srinivasan A. (1998). Two CD95 (APO-1/Fas) signaling pathways. *The EMBO Journal*.

[20] Chang H. Y., Nishitoh H., Yang X., Ichijo H., Baltimore D. (1998). Activation of Apoptosis signal-regulating kinase 1 (ASK1) by the adapter protein Daxx. *Science*.

[21] Paul A., Wilson S., Belham C. M. (1997). Stress-activated protein kinases: activation, regulation and function. *Cellular Signalling*.

[22] Yang X., Khosravi-Far R., Chang H. Y., Baltimore D. (1997). Daxx, a novel fas-binding protein that activates JNK and apoptosis. *Cell*.

[23] Raoul C., Pettmann B., Henderson C. E. (2000). Active killing of neurons during development and following stress: a role for p75(NTR) and Fas?. *Current Opinion in Neurobiology*.

[24] Ethell D. W., Buhler L. A. (2003). Fas ligand-mediated apoptosis in degenerative disorders of the brain. *Journal of Clinical Immunology*.

[25] Haase G., Pettmann B., Raoul C., Henderson C. E. (2008). Signaling by death receptors in the nervous system. *Current Opinion in Neurobiology*.

[26] Park C., Sakamaki K., Tachibana O., Yamashima T., Yamashita J., Yonehara S. (1998). Expression of Fas antigen in the normal mouse brain. *Biochemical and Biophysical Research Communications*.

[27] Raoul C., Buhler E., Sadeghi C. (2006). Chronic activation in presymptomatic amyotrophic lateral sclerosis (ALS) mice of a feedback loop involving Fas, Daxx, and FasL. *Proceedings of the National Academy of Sciences of the United States of America*.

[28] Zuliani C., Kleber S., Klussmann S. (2006). Control of neuronal branching by the death receptor CD95 (Fas/Apo-1). *Cell Death and Differentiation*.

[29] Raoul C., Estévez A. G., Nishimune H. (2002). Motoneuron death triggered by a specific pathway downstream of fas: potentiation by ALS-linked SOD1 mutations. *Neuron*.

[30] Franco M. C., Ye Y., Refakis C. A. (2013). Nitration of Hsp90 induces cell death. *Proceedings of the National Academy of Sciences of the United States of America*.

[31] Bernard-Marissal N., Moumen A., Sunyach C. (2012). Reduced calreticulin levels link endoplasmic reticulum stress and fas-triggered cell death in motoneurons vulnerable to ALS. *The Journal of Neuroscience*.

[32] Duplan L., Bernard N., Casseron W. (2010). Collapsin response mediator protein 4a (CRMP4a) is upregulated in motoneurons of mutant SOD1 mice and can trigger motoneuron axonal degeneration and cell death. *The Journal of Neuroscience*.

[33] Bendotti C., Atzori C., Piva R. (2004). Activated p38MAPK is a novel component of the intracellular inclusions found in human amyotrophic lateral sclerosis and mutant SOD1 transgenic mice. *Journal of Neuropathology and Experimental Neurology*.

[34] Catania M. V., Aronica E., Yankaya B., Troost D. (2001). Increased expression of neuronal nitric oxide synthase spliced variants in reactive astrocytes of amyotrophic lateral sclerosis human spinal cord. *The Journal of Neuroscience*.

[35] Holasek S. S., Wengenack T. M., Kandimalla K. K. (2005). Activation of the stress-activated MAP kinase, p38, but not JNK in cortical motor neurons during early presymptomatic stages of amyotrophic lateral sclerosis in transgenic mice. *Brain Research*.

[36] Phul R. K., Shaw P. J., Ince P. G., Smith M. E. (2000). Expression of nitric oxide synthase isoforms in spinal cord in amyotrophic lateral sclerosis. *Amyotrophic Lateral Sclerosis*.

[37] Ranganathan S., Williams E., Ganchev P. (2005). Proteomic profiling of cerebrospinal fluid identifies biomarkers for amyotrophic lateral sclerosis. *Journal of Neurochemistry*.

[38] Veglianese P., Lo Coco D., Bao Cutrona M. (2006). Activation of the p38MAPK cascade is associated with upregulation of TNF alpha receptors in the spinal motor neurons of mouse models of familial ALS. *Molecular and Cellular Neuroscience*.

[39] Wengenack T. M., Holasek S. S., Montano C. M., Gregor D., Curran G. L., Poduslo J. F. (2004). Activation of programmed cell death markers in ventral horn motor neurons during early presymptomatic stages of amyotrophic lateral sclerosis in a transgenic mouse model. *Brain Research*.

[40] Ahn S. W., Kim J. E., Park K. S. (2012). The neuroprotective effect of the GSK-3beta inhibitor and influence on the extrinsic apoptosis in the ALS transgenic mice. *Journal of the Neurological Sciences*.

[41] Almer G., Vukosavic S., Romero N., Przedborski S. (1999). Inducible nitric oxide synthase up-regulation in a transgenic mouse model of familial amyotrophic lateral sclerosis. *Journal of Neurochemistry*.

[42] Sasaki S., Warita H., Abe K., Iwata M. (2001). Inducible nitric oxide synthase (iNOS) and nitrotyrosine immunoreactivity in the spinal cords of transgenic mice with a G93A mutant SOD1 gene. *Journal of Neuropathology and Experimental Neurology*.

[43] Sasaki S., Shibata N., Komori T., Iwata M. (2000). iNOS and nitrotyrosine immunoreactivity in amyotrophic lateral sclerosis. *Neuroscience Letters*.

[44] Haidet-Phillips A. M., Hester M. E., Miranda C. J. (2011). Astrocytes from familial and sporadic ALS patients are toxic to motor neurons. *Nature Biotechnology*.

[45] Nagai M., Re D. B., Nagata T. (2007). Astrocytes expressing ALS-linked mutated SOD1 release factors selectively toxic to motor neurons. *Nature Neuroscience*.

[46] Barbeito L. H., Pehar M., Cassina P. (2004). A role for astrocytes in motor neuron loss in amyotrophic lateral sclerosis. *Brain Research Reviews*.

[47] Xiao Q., Zhao W., Beers D. R. (2007). Mutant SOD1G93A microglia are more neurotoxic relative to wild-type microglia. *Journal of Neurochemistry*.

[48] Ciesielski-Treska J., Ulrich G., Chasserot-Golaz S. (2001). Mechanisms underlying neuronal death induced by chromogranin A-activated microglia. *The Journal of Biological Chemistry*.

[49] Terrazzino S., Bauleo A., Baldan A., Leon A. (2002). Peripheral LPS administrations up-regulate Fas and FasL on brain microglial cells: a brain protective or pathogenic event?. *Journal of Neuroimmunology*.

[50] Sengun I. S., Appel S. H. (2003). Serum anti-Fas antibody levels in amyotrophic lateral sclerosis. *Journal of Neuroimmunology*.

[51] Yi F. H., Lautrette C., Vermot-Desroches C. (2000). In vitro induction of neuronal apoptosis by anti-Fas antibody-containing sera from amyotrophic lateral sclerosis patients. *Journal of Neuroimmunology*.

[52] Chiu I. M., Chen A., Zheng Y. (2008). T lymphocytes potentiate endogenous neuroprotective inflammation in a mouse model of ALS. *Proceedings of the National Academy of Sciences of the United States of America*.

[53] Liblau R. S., Gonzalez-Dunia D., Wiendl H., Zipp F. (2013). Neurons as targets for T cells in the nervous system. *Trends in Neurosciences*.

[54] Shao H., He Y., Li K. C., Zhou X. (2013). A system mathematical model of a cell-cell communication network in amyotrophic lateral sclerosis. *Molecular BioSystems*.

[55] Petri S., Kiaei M., Wille E., Calingasan N. Y., Beal M. F. (2006). Loss of Fas ligand-function improves survival in G93A-transgenic ALS mice. *Journal of the Neurological Sciences*.

[56] Locatelli F., Corti S., Papadimitriou D. (2007). Fas small interfering RNA reduces motoneuron death in amyotrophic lateral sclerosis mice. *Annals of Neurology*.

[57] Kiaei M., Kipiani K., Calingasan N. Y. (2007). Matrix metalloproteinase-9 regulates TNF-*α* and FasL expression in neuronal, glial cells and its absence extends life in a transgenic mouse model of amyotrophic lateral sclerosis. *Experimental Neurology*.

[58] Dewil M., Schurmans C., Starckx S., Opdenakker G., van den Bosch L., Robberecht W. (2005). Role of matrix metalloproteinase-9 in a mouse model for amyotrophic lateral sclerosis. *NeuroReport*.

[59] Lorenzl S., Narr S., Angele B. (2006). The matrix metalloproteinases inhibitor Ro 26-2853 extends survival in transgenic ALS mice. *Experimental Neurology*.

[60] Mauri D. N., Ebner R., Montgomery R. I. (1998). LIGHT, a new member of the TNF superfamily, and lymphotoxin *α* are ligands for herpesvirus entry mediator. *Immunity*.

[61] Scheu S., Alferink J., Pötzel T., Barchet W., Kalinke U., Pfeffer K. (2002). Targeted disruption of LIGHT causes defects in costimulatory T cell activation and reveals cooperation with lymphotoxin *β* in mesenteric lymph node genesis. *Journal of Experimental Medicine*.

[62] Tamada K., Shimozaki K., Chapoval A. I. (2000). LIGHT, a TNF-like molecule, costimulates T cell proliferation and is required for dendritic cell-mediated allogeneic T cell response. *Journal of Immunology*.

[63] Kwon B. S., Tan K. B., Ni J. (1997). A newly identified member of the tumor necrosis factor receptor superfamily with a wide tissue distribution and involvement in lymphocyte activation. *The Journal of Biological Chemistry*.

[64] Montgomery R. I., Warner M. S., Lum B. J., Spear P. G. (1996). Herpes simplex virus-1 entry into cells mediated by a novel member of the TNF/NGF receptor family. *Cell*.

[65] Chen M., Hsu T., Luh T., Hsieh S. (2000). Overexpression of Bcl-2 enhances LIGHT- and interferon-*γ*-mediated apoptosis in Hep3BT2 cells. *The Journal of Biological Chemistry*.

[66] Li J., Shen F., Wu D. (2007). Expression level of Bcl-XL critically affects sensitivity of hepatocellular carcinoma cells to LIGHT-enhanced and interferon-gamma-induced apoptosis. *Oncology Reports*.

[67] Rooney I. A., Butrovich K. D., Glass A. A. (2000). The lymphotoxin-*β* receptor is necessary and sufficient for LIGHT-mediated apoptosis of tumor cells. *The Journal of Biological Chemistry*.

[68] Wu M., Wang P., Han S., Hsieh S. (1999). The cytoplasmic domain of the lymphotoxin-*β* receptor mediates cell death in HeLa cells. *The Journal of Biological Chemistry*.

[69] Gavaldà N., Gutierrez H., Davies A. M. (2009). Developmental regulation of sensory neurite growth by the tumor necrosis factor superfamily member LIGHT. *The Journal of Neuroscience*.

[70] Plant S. R., Iocca H. A., Wang Y. (2007). Lymphotoxin *β* receptor (Lt*β*R): Dual roles in demyelination and remyelination and successful therapeutic intervention using Lt*β*R-Ig protein. *The Journal of Neuroscience*.

[71] Aebischer J., Cassina P., Otsmane B. (2011). IFN*γ* triggers a LIGHT-dependent selective death of motoneurons contributing to the non-cell-autonomous effects of mutant SOD1. *Cell Death and Differentiation*.

[72] Babu G. N., Kumar A., Chandra R., Puri S. K., Kalita J., Misra U. K. (2008). Elevated inflammatory markers in a group of amyotrophic lateral sclerosis patients from northern India. *Neurochemical Research*.

[73] Henkel J. S., Beers D. R., Wen S. (2013). Regulatory T-lymphocytes mediate amyotrophic lateral sclerosis progression and survival. *EMBO Molecular Medicine*.

[74] Hensley K., Fedynyshyn J., Ferrell S. (2003). Message and protein-level elevation of tumor necrosis factor *α* (TNF*α*) and TNF*α*-modulating cytokines in spinal cords of the G93A-SOD1 mouse model for amyotrophic lateral sclerosis. *Neurobiology of Disease*.

[75] Tateishi T., Yamasaki R., Tanaka M. (2010). CSF chemokine alterations related to the clinical course of amyotrophic lateral sclerosis. *Journal of Neuroimmunology*.

[76] Benveniste E. N., Benos D. J. (1995). TNF-*α*- and IFN-*γ*-mediated signal transduction pathways: effects on glial cell gene expression and function. *The FASEB Journal*.

[77] Aebischer J., Moumen A., Sazdovitch V., Seilhean D., Meininger V., Raoul C. (2012). Elevated levels of IFN*γ* and LIGHT in the spinal cord of patients with sporadic amyotrophic lateral sclerosis. *European Journal of Neurology*.

[78] Brahmachari S., Fung Y. K., Pahan K. (2006). Induction of glial fibrillary acidic protein expression in astrocytes by nitric oxide. *The Journal of Neuroscience*.

[79] Kreutzberg G. W. (1996). Microglia: a sensor for pathological events in the CNS. *Trends in Neurosciences*.

[80] Badie B., Schartner J., Vorpahl J., Preston K. (2000). Interferon-*γ* induces apoptosis and augments the expression of Fas and Fas ligand by microglia in vitro. *Experimental Neurology*.

[81] Cabal-Hierro L., Lazo P. S. (2012). Signal transduction by tumor necrosis factor receptors. *Cellular Signalling*.

[82] Elliott J. L. (2001). Cytokine upregulation in a murine model of familial amyotrophic lateral sclerosis. *Molecular Brain Research*.

[83] Hensley K., Floyd R. A., Gordon B. (2002). Temporal patterns of cytokine and apoptosis-related gene expression in spinal cords of the G93A-SOD1 mouse model of amyotrophic lateral sclerosis. *Journal of Neurochemistry*.

[84] Yoshihara T., Ishigaki S., Yamamoto M. (2002). Differential expression of inflammation- and apoptosis-related genes in spinal cords of a mutant SOD1 transgenic mouse model of familial amyotrophic lateral sclerosis. *Journal of Neurochemistry*.

[85] Cereda C., Baiocchi C., Bongioanni P. (2008). TNF and sTNFR1/2 plasma levels in ALS patients. *Journal of Neuroimmunology*.

[86] Poloni M., Facchetti D., Mai R. (2000). Circulating levels of tumour necrosis factor-*α* and its soluble receptors are increased in the blood of patients with amyotrophic lateral sclerosis. *Neuroscience Letters*.

[87] Gowing G., Dequen F., Soucy G., Julien J. (2006). Absence of tumor necrosis factor-*α* does not affect motor neuron disease caused by superoxide dismutase 1 mutations. *The Journal of Neuroscience*.

[88] Weydt P., Yuen E. C., Ransom B. R., Möller T. (2004). Increased cytotoxic potential of microglia from ALS-transgenic mice. *Glia*.

[89] Gowing G., Lalancette-Hébert M., Audet J., Dequen F., Julien J. (2009). Macrophage colony stimulating factor (M-CSF) exacerbates ALS disease in a mouse model through altered responses of microglia expressing mutant superoxide dismutase. *Experimental Neurology*.

[90] Dawbarn D., Allen S. J. (2003). Neurotrophins and neurodegeneration. *Neuropathology and Applied Neurobiology*.

[91] Ernfors P., Henschen A., Olson L., Persson H. (1989). Expression of nerve growth factor receptor mRNA is developmentally regulated and increased after axotomy in rat spinal cord motoneurons. *Neuron*.

[92] Koliatsos V. E., Crawford T. O., Price D. L. (1991). Axotomy induces nerve growth factor receptor immunoreactivity in spinal motor neurons. *Brain Research*.

[93] Ferri C. C., Moore F. A., Bisby M. A. (1998). Effects of facial nerve injury on mouse motoneurons lacking the p75 low-affinity neurotrophin receptor. *Journal of Neurobiology*.

[94] Lowry K. S., Murray S. S., McLean C. A. (2001). A potential role for the p75 low-affinity neurotrophin receptor in spinal motor neuron degeneration in murine and human amyotrophic lateral sclerosis. *Amyotrophic Lateral Sclerosis and Other Motor Neuron Disorders*.

[95] Seeburger J. L., Tarras S., Natter H., Springer J. E. (1993). Spinal cord motoneurons express p75(NGFR) and p145(trkB) mRNA in amyotrophic lateral sclerosis. *Brain Research*.

[96] Pehar M., Cassina P., Vargas M. R. (2004). Astrocytic production of nerve growth factor in motor neuron apoptosis: implications for amyotrophic lateral sclerosis. *Journal of Neurochemistry*.

[97] Pehar M., Vargas M. R., Robinson K. M. (2007). Mitochondrial superoxide production and nuclear factor erythroid 2-related factor 2 activation in p75 neurotrophin receptor-induced motor neuron apoptosis. *The Journal of Neuroscience*.

[98] Cassina P., Pehar M., Vargas M. R. (2005). Astrocyte activation by fibroblast growth factor-1 and motor neuron apoptosis: implications for amyotrophic lateral sclerosis. *Journal of Neurochemistry*.

[99] Küst B. M., Brouwer N., Mantingh I. J., Boddeke H. W. G. M., Copray J. C. V. M. (2003). Reduced p75NTR expression delays disease onset only in female mice of a transgenic model of familial amyotrophic lateral sclerosis. *Amyotrophic Lateral Sclerosis and other Motor Neuron Disorders*.

[100] Turner B. J., Cheah I. K., Macfarlane K. J. (2003). Antisense peptide nucleic acid-mediated knockdown of the p75 neurotrophin receptor delays motor neuron disease in mutant SOD1 transgenic mice. *Journal of Neurochemistry*.

[101] Ralph G. S., Radcliffe P. A., Day D. M. (2005). Silencing mutant SOD1 using RNAi protects against neurodegeneration and extends survival in an ALS model. *Nature Medicine*.

[102] Raoul C., Abbas-Terki T., Bensadoun J. (2005). Lentiviral-mediated silencing of SOD1 through RNA interference retards disease onset and progression in a mouse model of ALS. *Nature Medicine*.

[103] Mosley R. L., Gendelman H. E. (2010). Control of neuroinflammation as a therapeutic strategy for amyotrophic lateral sclerosis and other neurodegenerative disorders. *Experimental Neurology*.

[104] Kiaei M., Petri S., Kipiani K. (2006). Thalidomide and lenalidomide extend survival in a transgenic mouse model of amyotrophic lateral sclerosis. *The Journal of Neuroscience*.

[105] Neymotin A., Petri S., Calingasan N. Y. (2009). Lenalidomide (Revlimid) administration at symptom onset is neuroprotective in a mouse model of amyotrophic lateral sclerosis. *Experimental Neurology*.

[106] Meyer T., Maier A., Borisow N. (2008). Thalidomide causes sinus bradycardia in ALS. *Journal of Neurology*.

[107] Stommel E. W., Cohen J. A., Fadul C. E. (2009). Efficacy of thalidomide for the treatment of amyotrophic lateral sclerosis: a phase II open label clinical trial. *Amyotrophic Lateral Sclerosis*.

[108] Dunston C. R., Griffiths H. R., Lambert P. A., Staddon S., Vernallis A. B. (2011). Proteomic analysis of the anti-inflammatory action of minocycline. *Proteomics*.

[109] Gordon P. H., Moore D. H., Miller R. G. (2007). Efficacy of minocycline in patients with amyotrophic lateral sclerosis: a phase III randomised trial. *The Lancet Neurology*.

[110] Zhu S., Stavrovskaya I. G., Drozda M. (2002). Minocycline inhibits cytochrome c release and delays progression of amyotrophic lateral sclerosis in mice. *Nature*.

[111] Cudkowicz M. E., Shefner J. M., Schoenfeld D. A. (2006). Trial of celecoxib in amyotrophic lateral sclerosis. *Annals of Neurology*.

[112] Drachman D. B., Frank K., Dykes-Hoberg M. (2002). Cyclooxygenase 2 inhibition protects motor neurons and prolongs survival in a transgenic mouse model of ALS. *Annals of Neurology*.

[113] Dong Y., Benveniste E. N. (2001). Immune function of astrocytes. *Glia*.

[114] Henkel J. S., Beers D. R., Zhao W., Appel S. H. (2009). Microglia in ALS: the good, the bad, and the resting. *Journal of Neuroimmune Pharmacology*.

[115] Moisse K., Strong M. J. (2006). Innate immunity in amyotrophic lateral sclerosis. *Biochimica et Biophysica Acta*.

[116] Glass C. K., Saijo K., Winner B., Marchetto M. C., Gage F. H. (2010). Mechanisms underlying inflammation in neurodegeneration. *Cell*.

